# Simulating and Evaluating Local Interventions to Improve Cardiovascular Health

**Published:** 2009-12-15

**Authors:** Jack Homer, Bobby Milstein, Darwin Labarthe, Diane Orenstein, Kristina Wile, Justin Trogdon, Philip Huang

**Affiliations:** Homer Consulting; Centers for Disease Control and Prevention, Atlanta, Georgia; Centers for Disease Control and Prevention, Atlanta, Georgia; Centers for Disease Control and Prevention, Atlanta, Georgia; Sustainability Institute, Stow, Massachusetts; RTI International, Research Triangle Park, North Carolina; Austin/Travis County Health and Human Services Department, Austin, Texas

## Abstract

Numerous local interventions for cardiovascular disease are available, but resources to deliver them are limited. Identifying the most effective interventions is challenging because cardiovascular risks develop through causal pathways and gradual accumulations that defy simple calculation. We created a simulation model for evaluating multiple approaches to preventing and managing cardiovascular risks. The model incorporates data from many sources to represent all US adults who have never had a cardiovascular event. It simulates trajectories for the leading direct and indirect risk factors from 1990 to 2040 and evaluates 19 interventions. The main outcomes are first-time cardiovascular events and consequent deaths, as well as total consequence costs, which combine medical expenditures and productivity costs associated with cardiovascular events and risk factors. We used sensitivity analyses to examine the significance of uncertain parameters. A base case scenario shows that population turnover and aging strongly influence the future trajectories of several risk factors. At least 15 of 19 interventions are potentially cost saving and could reduce deaths from first cardiovascular events by approximately 20% and total consequence costs by 26%. Some interventions act quickly to reduce deaths, while others more gradually reduce costs related to risk factors. Although the model is still evolving, the simulated experiments reported here can inform policy and spending decisions.

## Introduction

Conditions in particular neighborhoods or cities can profoundly enhance or impede people's prospects for a healthy life (1). This dependence on local context is especially evident in cardiovascular health, for which behavioral, social, and environmental factors combine to affect the likelihood of developing disease or dying prematurely (2). Heart disease and stroke are largely preventable, but they remain the first and third leading causes of death in the United States, partly because we have yet to establish living conditions that minimize such modifiable risks as smoking, obesity, stress, air pollution, poor diet, and physical inactivity. The importance of intervening to limit these risks is highlighted in *A Public Health Action Plan to Prevent Heart Disease and Stroke* (3).

The notion that cardiovascular disease (CVD) can be prevented through local actions raises practical questions that can be examined through systems modeling and simulation. Working closely with colleagues in Austin/Travis County, Texas, and subject matter experts at the Centers for Disease Control and Prevention and the National Institutes of Health, we developed a system dynamics simulation model to answer the following questions:

- How does local context affect the major risk factors for CVD and, in turn, population health status and costs?
- How might local interventions affect CVD risk, health status, and costs over time?
- How might local health leaders better balance their policy efforts given limited resources?

## Methods

System dynamics models improve our ability to anticipate the likely effects of interventions in dynamically complex situations, where the pathways from interventions to outcomes may be indirect, delayed, and possibly affected by nonlinearities or feedback loops (4). System dynamics has been used effectively since the 1970s to model many areas of public health and social policy, including CVD (5).

### Model structure

**Figure 1:**
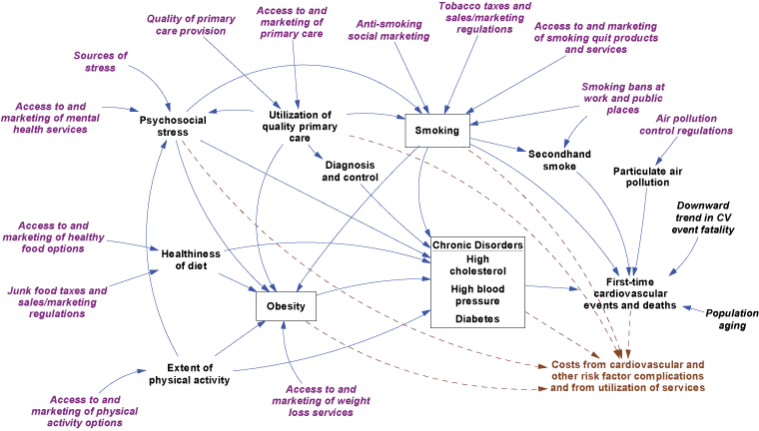
Simulation model for cardiovascular disease (CVD) outcomes. This diagram depicts major health conditions related to CVD and their causes. Boxes identify risk factor prevalence rates modeled as dynamic stocks. The population flows associated with these stocks — including people entering the adult population, entering the next age category, immigration, risk factor incidence, recovery, cardiovascular event survival, and death — are not shown.

We previously described a framework for understanding cardiovascular health in a local context (6). That framework has been refined and quantified by using additional literature and input from veteran health planners and analysts. The resulting simulation model (Figure 1) focuses on primary prevention; it does not address people who have experienced a CVD event. Causal influences move down and to the right, ending with 2 outcomes: 1) first-time cardiovascular events and consequent deaths and 2) costs associated with these events and with the identified risk factors.

**Table:** 

**Key:**
Blue solid arrows: causal linkages affecting risk factors and cardiovascular events and deaths.
Brown dashed arrows: influences on costs.
Purple italics: factors amenable to direct intervention.
Black italics (population aging, cardiovascular event fatality): other specified trends.
Black nonitalics: all other variables, affected by italicized variables and by each other.

**Table:** 

This diagram consists of word phrases (representing model variables) connected by one-way arrows (representing causal linkages). These words and arrows are in different colors and styles whose meaning is explained in the title and key accompanying the figure.
In the center of the diagram are several variables in plain black text, some in boxes representing dynamic stocks and all having both incoming and outgoing arrows. Proceeding from top to bottom and left to right, these variables are “psychosocial stress,” “healthiness of diet,” “extent of physical activity,” “utilization of quality primary care,” “diagnosis and control,” “obesity” (in a box), “smoking” (in a box), “chronic disorders” (with “high cholesterol,” “high blood pressure,” and “diabetes,” together in a box), “secondhand smoke,” “particulate air pollution,” and “first-time cardiovascular events and deaths.”
Concepts in purple italic text (representing interventions) lie around the top and left edges of the diagram, having no arrows coming into them, but all having outgoing blue arrows linking into the plain black text variables listed above. The links (moving clockwise from lower left to upper right) are as follows: “Access to and marketing of weight loss services” links to “obesity.” “Access to and marketing of physical activity options” links to “extent of physical activity.” “Access to and marketing of healthy food options” as well as “junk food taxes and sales/marketing regulations” link to “healthiness of diet.” “Access to and marketing of mental health services” and “sources of stress” link to “psychosocial stress.” “Quality of primary care provision” and “Access to and marketing of primary care” link to “utilization of quality primary care.” “Anti-smoking social marketing,” “tobacco taxes and sales/marketing regulations,” “access to and marketing of smoking quit products and services,” and “smoking bans at work and public places” link to “smoking.” “Smoking bans at work and public places” also links to “secondhand smoke.” “Air pollution control regulations” links to “particulate air pollution.”
Blue-arrow links also indicate causal connections among the plain black text variables. The links are as follows: “Extent of physical activity” and “utilization of quality primary care” link to “psychosocial stress.” “Utilization of quality primary care” links to “diagnosis and control.” “Psychosocial stress,” “healthiness of diet,” “extent of physical activity,” “utilization of quality primary care,” and “smoking” link to the “obesity” box. “Utilization of quality primary care” and “psychosocial stress” link to the “smoking” box. “Psychosocial stress,” “healthiness of diet,” “extent of physical activity,” “diagnosis and control,” “obesity,” and “smoking” link to the “chronic disorders” box. “Smoking” links to “secondhand smoke.” “Smoking,” “chronic disorders,” “secondhand smoke, and “particulate air pollution” link to “first-time cardiovascular events and deaths.”
To the right of “first-time cardiovascular events and deaths” and leading to it with blue arrows are 2 variables in black italic text with no incoming arrows: “downward trend in CV event fatality” and “population aging.”
In the lower right corner of the diagram, in plain brown text, is “costs from cardiovascular and other risk factor complications and from utilization of services.” Leading to this variable are brown arrows from “psychosocial stress,” “obesity,” “utilization of quality primary care,” “smoking,” “chronic disorders,” and “first-time cardiovascular events and deaths.”

The model starts with conditions in the United States in 1990 and simulates them continuously through 2040. The population without CVD and risk factor prevalence rates are represented as dynamic stock or state variables, subdivided by sex and age group (18-29 y, 30-64 y, and ≥65 y). Smoking and obesity are viewed as reversible conditions, whereas diabetes, high blood pressure, and high cholesterol are viewed as chronic conditions that are not reversible but that can be controlled, with the help of good-quality primary health care, to reduce CVD risk.

The incidence of first-time CVD events in the model is driven by the effect of several direct risk factors, based on a widely used risk calculator from the Framingham Heart Study (7). We modified that calculator in several ways for this study, most fundamentally by estimating annual risks at a population level on the basis of risk factor prevalence rates, rather than at the level of an individual. (A detailed description of the modified calculator is available at http://sustainer.org/cvd/documents/SELI_App1.pdf). We also recognized the direct effect on CVD events, especially myocardial infarctions, from secondhand smoke and small particulate matter (PM 2.5) air pollution (8-11). Furthermore, because deaths from CVD have declined partly because of improvements in emergency and acute care, we incorporated a downward trend in the CVD case-fatality rate for 1990 through 2003 (12).

Obesity contributes to CVD, largely through diabetes, high blood pressure, and high cholesterol levels (13). Other indirect influences in the model are physical inactivity, poor diet, psychosocial stress, and smoking as it affects diabetes and obesity (14-21).

Both the direct and indirect influences in the model may be modified by 19 interventions (Table 1). These 19 interventions could be implemented at a city, county, or state level rather than requiring changes nationwide. In functional terms, the interventions are of a few basic types: those that provide broader access to health-promoting services, those that promote desirable behaviors, and those that tax or regulate to deter undesirable behaviors.

### C**ost calculation**

We used a common metric — constant 2005 dollars — to track medical and productivity costs (for morbidity and mortality) that might be affected by the 19 interventions. We measured the societal value of morbidity (sick days) and premature mortality (years of life lost) using a human capital approach, which estimates the market value of lost productivity at work and at home (22). (A detailed description of cost calculations is available at http://sustainer.org/cvd/documents/SELI_App2.pdf.) This summary of medical and productivity costs can determine whether any intervention, or package of interventions, is justified by its likely aggregate consequences, or "total consequence costs." We did not estimate the costs of interventions. However, the total consequence costs can inform spending decisions. For example, suppose that for a given intervention the model calculates a total consequence cost savings of $50 per capita. Planners may then conclude that up to $50 per capita could be justifiably spent to implement that intervention and still create positive net benefits to society.

The model tracks 3 types of intervention consequences:

- Medical and productivity costs attributable to fatal and nonfatal CVD events.
- Medical and productivity costs attributable to noncardiovascular complications of smoking (eg, lung cancer), diabetes (eg, blindness), high blood pressure (eg, kidney failure), and obesity (eg, colorectal cancer). We have thus far been able to quantify these costs, but not yet the costs related to noncardiovascular complications of stress (eg, depression), physical inactivity (eg, back pain), or poor diet (eg, colorectal cancer).
- Costs of services and products to manage risk factors. These include medications and visits for managing chronic disorders, mental health services, weight-loss services, and smoking cessation services and products.

### Model calibration

Although the model is meant to investigate interventions in localities such as Austin/Travis County, we began by calibrating it to represent the entire United States. This approach enabled more precise estimation, given that certain data were either unreliable or unavailable at the local level. The results are generally reported as per capita estimates to facilitate interpretation at a local level. Table 2 lists the major information sources on which the model is based (23-32).

The model specifies initial (1990) incidence rates for smoking, obesity, diabetes, high blood pressure, and high cholesterol, as well as cessation rates for smoking and obesity. These parameters have been set so that the model accurately simulates the observed changes in prevalence rates in the National Health and Nutrition Examination Survey from 1988-1994 to 1999-2004.

The model also contains 56 causal links requiring the estimation of relative risks, effect sizes, or initial values. Many of these parameters were estimated through the use of published studies, meta-analyses, and in some instances, ad hoc surveys of veteran practitioners (33). Because most of these parameter estimates have some level of uncertainty, we also identified lower and upper bounds to be used for sensitivity analysis.

### Model testing

Having calibrated the model to accurately reproduce observed trends in risk factor prevalence rates as well as CVD events and deaths, we then explored plausible futures. A base case scenario assumed no changes after 2004 in many of the local determinants of risk in the adult population, including healthiness of diet, extent of physical activity, stress, use of quality primary care, air pollution, and the prevalence rates of smoking and obesity among incoming 18-year-olds. This base case should not be taken as a statement about what is most likely to happen in the absence of intervention, but rather serves as a straightforward and easily understood benchmark against which to compare intervention scenarios.

We tested interventions singly and in groups of similar interventions. For all interventions, we assumed a 1-year ramp-up during 2009, followed by full implementation from 2010 through 2040. The significance of full implementation depends on the intervention, but in all cases is based on effect sizes that the research literature or veteran practitioners indicate should be possible:

- For the 7 marketing interventions and for taxes on tobacco or junk food: doing the maximum that has been demonstrated or seriously proposed somewhere in the United States.
- For the 6 access interventions: raising access to 100%.
- For smoking restriction: reducing secondhand exposures in workplaces and public places to zero.
- For air pollution: reducing small particulate matter by 50% from its 2001-2003 value.
- For sources of chronic stress: a 50% reduction.
- For the quality of primary care (ie, adherence to guidelines): improvement from a national average of 54% (27) to 75%.

For each intervention scenario, we conducted separate simulations using the midpoint, lower-end, and upper-end values for all uncertain parameters. This method yielded a range of plausible outcomes for each intervention scenario.

## Discussion

### Base case results

The base case projects that even after 2004, when we assume no further changes to the model's inputs, historical trends in the model's risk factor prevalence rates will continue through 2040, although with diminishing slopes. In particular, the model projects further declines in smoking (and, thus, secondhand smoke exposure) and high cholesterol, and at the same time further growth in high blood pressure and diabetes. The projected continuation of past trends reflects the eventual death of older cohorts and their replacement by younger cohorts with different habits and characteristics. For instance, the continued decline of smoking prevalence reflects the lower rate of smoking among teens and young adults today than in previous decades. Such demographic turnover also helps explain the continued growth of high blood pressure and diabetes, which occurs in the model as a legacy of the increase in obesity — a leading risk factor for both disorders — from 1980 to 2004. The projected continuation of trends also reflects the future aging of the population; the over-65 population will increase from 2010 through 2030. This aging effect contributes to the projected decline in smoking because smoking is much less common among the elderly. It also contributes to the projected increase in high blood pressure and diabetes because the prevalence of these disorders is higher with increasing age.

Deaths from first-time CVD events, which declined by 35% from 1990 to 2004, are projected in the base case to rebound by 33% from 2004 to 2040. Much of the past decline is attributable to a 28% reduction in the event fatality rate, from improvements in emergency and acute care. But it also reflects an 11% decline in the rate of CVD events that occurred, despite increases in high blood pressure and diabetes, because of decreases in smoking, secondhand smoke, PM 2.5 air pollution, and uncontrolled high cholesterol.

The potential future rebound in deaths anticipated by our model reflects a 17% increase in fatality from CVD events per capita and a 15% increase in the rate of CVD events because of the aging of the population. Although the base case projects no future increase in CVD events or deaths within each age group, the aging of the population will lead to an increase in the overall rate of CVD events and deaths.

Per capita total consequence costs, which the model calculates to have declined by 25% from 1990 to 2004, are projected in the base case to decline by another 5% from 2004 to 2040. Total consequence costs encompass not only CVD events (which account for 44% of the total costs in 2004) but also noncardiovascular complications of risk factors (also 44%) and management of risk factors (12%). Although per capita CVD event costs are projected to increase by 12% from 2004 to 2040 (reflecting the increase in the frequency of the events themselves) and per capita risk management costs are projected to increase by 8% (reflecting the growing demand for blood pressure and diabetes treatment), these increases are more than offset by a 25% decrease in noncardiovascular complications. This decrease is due to the projected decline of smoking, which in 2004 was responsible for more than 400,000 noncardiovascular deaths, primarily from lung cancer and chronic obstructive pulmonary disease. These premature non-CVD deaths from smoking account for a large fraction (about 28% in 2004) of the total consequence costs calculated in the model.

### Intervention scenario results

Individual tests of the 19 interventions suggest that each can reduce deaths from first-time CVD events, and most can reduce total consequence costs. Four of the interventions, however, raise total consequence costs, meaning that they increase risk factor management costs more than they decrease the costs of medical events and complications. These 4 interventions include the 2 that encourage use of mental health services and the 2 that encourage use of weight-loss services. However, because of limitations in the model, planners should not dismiss these interventions in the real world. In the case of mental health services, we have not yet estimated the noncardiovascular costs of depression. In the case of weight-loss services for obese people, our estimates of cost and benefit are based on conventional dieting and exercise programs, rather than on bariatric surgery, which, although more costly, also appears to be more effective (34).

**Figure 2:**
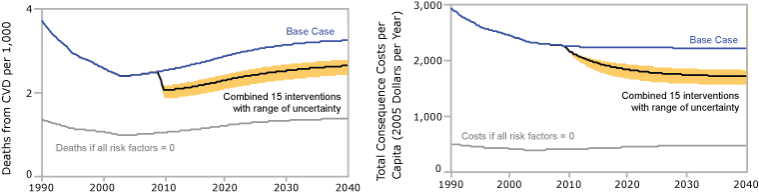
Estimated impacts of a 15-component intervention, with ranges based on sensitivity testing, simulation model for cardiovascular disease (CVD) outcomes. The 15 interventions are listed in Table 1 under the topical clusters of Care, Air, and Lifestyle.

We present simulation results for only the 15 interventions that in the model do not increase total consequence costs (Figure 2). Such a multipronged approach may be challenging to implement, given resource limitations, but it is useful to look at what could be achieved.

**Table:** 

**Key:**
Blue line = base case results.
Black line = expected reduction in death rate or costs from the 15-component intervention when the uncertain parameters are all set to their baseline values.
Orange shaded area around the black line = envelope of plausible outcomes in the 15-component intervention outcomes based on sensitivity testing. Upper edge (least impact) results when all uncertain impact parameters are set to their lowest values, while lower edge (most impact) results when all uncertain impact parameters are set to their greatest values.
Gray line = the model’s calculation of what the death rate or costs would be if all of the risk factors in the model — smoking, small particulate matter (PM 2.5) air pollution, high blood pressure, high cholesterol, diabetes, obesity, poor nutrition, inactivity, and stress — were reduced to zero.

**Table:** 

This figure consists of 2 line graphs that portray simulated death rate and cost outcomes for a 15-component intervention, showing the mean trajectory and upper and lower ranges based on sensitivity.
The graph on the left has an X axis labeled with the years 1990 to 2040 and a Y axis labeled “Deaths from CVD per 1,000.” The Y axis ranges from zero to 4. A blue line indicates the base case, with a death rate that starts at 3.70 deaths per 1,000 in 1990, dips to a low point of 2.37 in 2003, and rises gradually to 3.23 in 2040. A black line indicates the lower death rate that results from a 15-component intervention, implemented in 2009, with uncertain parameters all set to their baseline values. It quickly drops to 2.04 deaths per 1,000 in 2010 (compared with 2.50 in the base case), then gradually rises to 2.62 in 2040. The black line is surrounded by an orange shaded area that indicates a range of plausible death rate outcomes for the 15-component intervention. The upper edge (least impact — all uncertain impact parameters set to their lowest values) begins at 2.14 deaths per 1,000 in 2010 and rises gradually to a rate of 2.77 in 2040. The lower edge (most impact — all uncertain impact parameters set to their highest values) begins at 1.87 in 2010 and rises gradually to 2.41 in 2040. A gray line indicates a CVD death rate if all risk factors are set to zero. It begins at a rate of 1.32 deaths per 1,000 in 1990, declines to a low point of 0.96 in 2003, and rises gradually to 1.35 in 2040.
The graph on the right has an X axis labeled with the years 1990 to 2040 and a Y axis labeled “Total Consequence Costs per Capita (2005 Dollars per Year).” The Y axis ranges from zero to 3,000. A blue line indicates the base case, with a consequence cost per capita that starts at 2,934 in 1990, declines to 2,304 in 2004, and continues declining more gradually to 2,196 in 2040. A black line indicates the lower cost that results from the 15-component intervention, implemented in 2009, with uncertain parameters all set to their baseline values. It declines to 1,756 in 2020 (compared with 2,211 in the base case), then declines more gradually to 1,631 in 2040. The black line is surrounded by an orange shaded area that indicates a range of plausible cost outcomes for the 15-component intervention. The upper edge (least impact — all uncertain impact parameters set to their lowest values) declines to 1,883 in 2020 and 1,780 in 2040, while the lower edge (most impact — all uncertain impact parameters set to their greatest values) declines to 1,575 in 2020 and 1,474 in 2040. A gray line indicates the cost if all risk factors are set to zero. It starts in 1990 at 476, declines slightly to a low point of 371 in 2003, and rises slightly to 455 in 2040.

The model suggests that if all risk factors in the model were eliminated, the death rate could be reduced by approximately 60% below the base case, which falls between the 50% to 75% rate that other authors have suggested (35). This model dichotomizes blood pressure, cholesterol, and diabetes as "high" or "not high" and does not further subdivide the "not high" into normal and borderline. Reducing borderline conditions (prehypertension, borderline cholesterol, prediabetes) to normal could further reduce CVD, but we cannot explore this possibility with this model. (A static analysis of the potential benefits of reducing both high and borderline conditions is available at http://sustainer.org/cvd/documents/SELI_App3.pdf.)

The model projects that a 15-component intervention could reduce the first-time CVD event death rate relative to the base case by 20% (range based on sensitivity analysis, 15%-26%) in 2015 and by 19% (range, 14%-25%) in 2040. Thus, the interventions that could reduce CVD deaths have a relatively rapid effect.

The effect of the interventions is more gradual with regard to total consequence costs than it is with regard to CVD deaths; nearly 40% of the eventual effect on costs occurs after 2015 (Figure 2). If all risk factors in the model were eliminated, consequence costs could be reduced by approximately 80% below the base case. Relative to the base run, the 15-component intervention reduces consequence costs by 16% (range, 12%-23%) in 2015, eventually reaching 26% (range, 19%-33%) in 2040. The reduction in consequence costs is $348 per capita (range, $254-$514) in 2015 and $565 per capita (range, $416-$722) in 2040.

**Figure 3:**
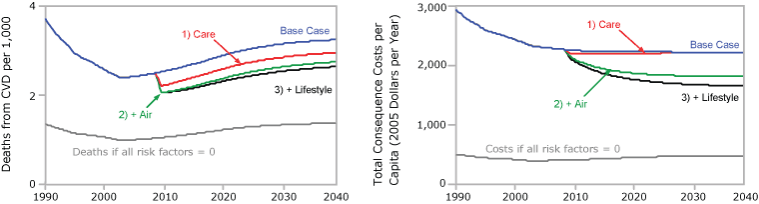
Projected changes in the death rate from first-time cardiovascular disease (CVD) events and in total consequence costs per capita when 15 interventions are combined, expressed in terms of clusters of interventions, simulation model for cardiovascular health outcomes.

The 15-component intervention may be better understood by examining the incremental contributions of its components grouped by topical cluster (Figure 3). We used the same base case graph as in Figure 2 and then incrementally added the following topical clusters:

1) The 3 interventions that improve the use and quality of primary care (Care).

2) The 6 interventions related to air quality and smoking (Air).

3) The 5 interventions related to improved nutrition and physical activity and the 1 intervention that would reduce sources of stress (Lifestyle).

**Table:** 

**Key:**
Blue line = base case results.
Gray line = outcomes if all risk factors were reduced to zero.
Red line = implement the 3 interventions that improve the use and quality of primary care (Care).
Green line = add the 6 interventions related to air quality and smoking (Air).
Black line = add the 5 interventions related to improved nutrition and physical activity and the 1 intervention that would reduce sources of stress (Lifestyle). This scenario includes all 15 interventions and is identical to the black line in Figure 2.

This figure consists of 2 line graphs that portray simulated death rate and cost outcomes for topical clusters of interventions, layered additively and building up to a 15-component intervention. All interventions are implemented in 2009. Three Care interventions improve the quality of primary care. Six Air interventions are related to air quality and smoking. Six Lifestyle interventions are related to improved nutrition and physical activity and reduced sources of stress.
The graph on the left has an X axis labeled with the years 1990 to 2040 and a Y axis labeled “Deaths from CVD per 1,000.” The Y axis ranges from zero to 4. A blue line indicates the base case, with a death rate that starts at 3.70 deaths per 1,000 in 1990, dips to a low point of 2.37 in 2003, and rises gradually to 3.23 in 2040. A red line indicates the effect of the Care cluster (3 interventions). It quickly drops to 2.18 deaths per 1,000 in 2010 (compared with 2.50 in the base case), then gradually rises to 2.93 in 2040. A green line indicates the effect of the combined Air and Care clusters (9 interventions). It quickly drops to 2.04 deaths per 1,000 in 2010, then gradually rises to 2.73 in 2040. A black line indicates the effect of the combined Air, Care, and Lifestyle clusters (15 interventions). It quickly drops to 2.04 deaths per 1,000 in 2010, then gradually rises to 2.62 in 2040. A gray line indicates a CVD death rate if all risk factors are set to zero. It begins at a rate of 1.32 deaths per 1,000 in 1990, declines to a low point of 0.96 in 2003, and rises gradually to 1.35 in 2040.
The graph on the right has an X axis labeled with the years 1990 to 2040 and a Y axis labeled “Total Consequence Costs per Capita (2005 Dollars per Year).” The Y axis ranges from zero to 3,000. A blue line indicates the base case, with a consequence cost per capita that starts at 2,934 in 1990, declines to 2,304 in 2004, and continues declining more gradually to 2,196 in 2040. A red line indicates the effect of the Care cluster (3 interventions). It declines only slightly to 2,184 in 2010, and then remains essentially flat or rising very slightly, ending at 2,189 in 2040. A green line indicates the effect of the combined Air and Care clusters (9 interventions). It declines to 1,859 in 2020 (compared with 2,211 in the base case), then declines more gradually to 1,796 in 2040. A black line indicates the effect of the combined Air, Care, and Lifestyle clusters (15 interventions). It declines to 1,756 in 2020, then declines more gradually to 1,631 in 2040. A gray line indicates the cost if all risk factors are set to zero. It starts in 1990 at 476, declines slightly to a low point of 371 in 2003, and rises slightly to 455 in 2040.

The relative effects of the clusters are different for CVD deaths than they are for total consequence costs. Of the 3 topical clusters, the largest contributor to projected reductions in CVD deaths, both in 2015 and 2040, is Care, followed by Air and then a smaller (though growing) contribution from Lifestyle. In contrast, the largest contributor to projected cost reductions, both in 2015 and 2040, is Air, followed by Lifestyle and then a smaller (and ultimately negligible) contribution from Care. The contributions to per capita cost reduction in 2015 are $235 from Air, $71 from Lifestyle, and $42 from Care. The contributions in 2040 are $393 from Air, $165 from Lifestyle, and $7 from Care.

## Conclusions

The major factors that affect cardiovascular health at a population level interact through causal pathways and develop through gradual accumulations that defy simple calculation. This dynamic complexity — and not just gaps in data — is a challenge for local leaders who want to intervene most effectively given limited resources. Our simulation model helps meet this challenge by integrating what is known about the various risk factors in a single testable framework for prospective policy analysis.

The simulations reported here point to several conclusions that local leaders and national allies may find valuable.

1) The CVD death rate has declined in recent years, not only because of improvements in emergency and acute care but also because of reductions in the CVD event rate itself, due to reductions in smoking, secondhand smoke, particulate air pollution, and uncontrolled high cholesterol. If this progress does not continue at a similar pace in the future, however, the CVD death rate will likely rebound strongly as the population ages.

2) Medical and productivity costs associated with CVD risk factors have declined because of declines in first-time CVD events and consequent deaths, and because of reductions in non-CVD deaths (especially lung cancer and chronic obstructive pulmonary disease) associated with smoking. Population aging will likely keep smoking prevalence on a path of decline into the future, so that even if CVD deaths rebound, the total consequence costs need not rebound.

3) Of 19 interventions that local planners may consider for lowering CVD risk, at least 15 could reduce CVD deaths without increasing total consequence costs.

4) Interventions aimed at reducing smoking and improving indoor and outdoor air quality can save lives relatively quickly and can justify intervention spending equivalent to as much as $300 per capita per year for 30 years (in 2005 constant dollars, without time discounting) to achieve the full implementation targets. Most local health leaders are already aware of the need for tobacco control and smoking bans, but many may not be aware of the contribution of particulate air pollution to CVD risk, even in areas like Austin/Travis County without heavy pollution.

5) Interventions aimed at improving the use and quality of primary care to diagnose and control high blood pressure, high cholesterol, and diabetes can save lives quickly but should not be expected to save much on total costs, primarily because of the high cost of medications. Consequently, the intervention spending to achieve and maintain such improvement should not exceed the equivalent of $25 per capita per year for 30 years. Other researchers have similarly found that good preventive care for chronic conditions may be cost-effective but is not necessarily cost-saving (36,37).

6) Interventions to improve nutrition and physical activity and to reduce sources of stress take more time to affect CVD deaths, as they gradually reduce obesity and other chronic disorders. Nonetheless, their contribution grows over time and may justify intervention spending equivalent to as much as $100 per capita per year for 30 years.

The ability of particular localities to achieve full implementation within these cost limits may vary depending on context and implementation factors. Potential extensions and improvements to the model include the following:

- Modeling medical and personal costs for the post-CVD event population and targeted interventions for secondary prevention to reduce the rate of recurrent CVD events.
- Modeling the prevalence rates of borderline conditions (prehypertension, borderline cholesterol, prediabetes) and incorporating them in the CVD risk calculations.
- Modeling the prevalence of former smokers and incorporating their differential risks in the CVD event and cost calculations.
- Incorporating the non-CVD consequences of stress, physical inactivity, and poor diet.
- Estimating intervention implementation costs to better inform intervention priorities.
- Incorporating additional independent risk factors for CVD (eg, excess sodium intake, excess trans fat intake, vitamin D deficiency, periodontal disease).

The model described here was created through a close collaboration with health planners in Austin/Travis County, who are now using a locally calibrated version of the model to support local strategy design and leadership development. We plan to pursue similar engagements with colleagues elsewhere. With more widespread use, this tool may help health planners across the country transform local contexts to most effectively improve cardiovascular health.

## Figures and Tables

**Table 1:** Interventions Used in Simulation Model for Cardiovascular Health Outcomes, Organized by Topical Cluster

**Topical Cluster**	**Intervention**
**Care**	Access to affordable primary care Promotion of primary care use Good-quality primary care
**Air**	Tobacco taxes and sales restrictions Social marketing against smoking Access to affordable smoking cessation services and products Promotion of smoking cessation Bans on smoking in public places Regulations and incentives that reduce air pollution
**Lifestyle**	Access to affordable healthy foods Promotion of healthy diet Junk food taxes and sales restrictions Access to safe and affordable physical activity Promotion of physical activity Reduced sources of chronic stress through improved living conditions and social supports
**Weight-loss and mental health services**	Access to affordable weight-loss services for the obese Promotion of weight-loss services Access to affordable mental health services for the chronically stressed Promotion of mental health services

**Table 2:** Information Sources Used in Simulation Model for Cardiovascular Health Outcomes

Topic	Source
Population size, growth, and aging, and health care coverage	US Census
Rates of cardiovascular events and deaths	Reports from American Heart Association (23) and National Institutes of Health (12)
Prevalence rates of smoking, obesity, and chronic disorders, and rates of diagnosis and control of chronic disorders	National Health and Nutrition Examination Survey (NHANES), 1988-1994 and 1999-2004
Fraction of 18-year-olds who smoke, are obese, or have chronic disorders	NHANES, Youth Risk Behavior Surveillance System
Prevalence of psychosocial stress	Behavioral Risk Factor Surveillance System (BRFSS)
Access to and use of good nutrition, physical activity, and primary care	BRFSS
Rates of smoking cessation	Mendez et al (24), Sloan et al (25)
Rates of people moving from obese to nonobese	Homer et al (26)
Trend in fraction of workplaces allowing smoking	Surgeon General's report (11)
Trend in small particulate matter (PM 2.5) air pollution	Dominici et al (9)
Average quality of primary care	Asch et al (27)
Medical costs, sick days, and years of life lost due to CVD events and deaths	Social Security actuarial life tables, Haddix et al (22), Russell et al (28), Sasser et al (29)
Noncardiovascular medical costs and sick days due to smoking, obesity, diabetes, and high blood pressure	Linked files of Medical Examination Panel Survey, National Health Interview Survey
Noncardiovascular mortality and years of life lost due to smoking, obesity, diabetes, and high blood pressure	Centers for Disease Control and Prevention Smoking-Attributable Mortality, Morbidity, and Economic Costs (SAMMEC), World Health Organization Statistical Information System, Flegal et al (30), American Diabetes Association (31), Clausen et al (32)

## References

[1] Bell J, Rubin V (2007). Why place matters: building a movement for healthy communities.

[2] Labarthe D (1998). Epidemiology and prevention of cardiovascular diseases: a global challenge.

[3] (2003). A public health action plan to prevent heart disease and stroke.

[4] Sterman JD (2000). Business dynamics: systems thinking and modeling for a complex world.

[5] Homer JB, Hirsch GB (2006). System dynamics modeling for public health: background and opportunities. Am J Public Health.

[6] Homer J, Milstein B, Wile K, Pratibhu P, Farris R, Orenstein D (2008). Modeling the local dynamics of cardiovascular health: risk factors, context, and capacity. Prev Chronic Dis.

[7] Anderson KM, Odell PM, Wilson PW, Kannel WB (1991). Cardiovascular disease risk profiles. Am Heart J.

[8] Pope CA, Burnett RT, Thurston GD, Thun MJ, Calle EE, Krewski D (2004). Cardiovascular mortality and long-term exposure to particulate air pollution: epidemiological evidence of general pathophysiological pathways of disease. Circulation.

[9] Dominici F, Peng RD, Zeger SL, White RH, Samet JM (2007). Particulate air pollution and mortality in the United States: did the risks change from 1987 to 2000?. Am J Epidemiol.

[10] Mowery PD, Babb S, Bishop EE, Pechacek TF Comparison of two methods for estimating prevalence of exposure to secondhand smoke in the US population. Paper presented at Towards a Smokefree Society Conference.

[11] (2006). The health consequences of involuntary exposure to tobacco smoke: a report of the Surgeon General.

[12] (2007). Morbidity and mortality: chart book on cardiovascular, lung, and blood diseases.

[13] Thompson D, Edelsberg J, Colditz GA, Bird AP, Oster G (1999). Lifetime health and economic consequences of obesity. Arch Intern Med.

[14] Katzmarzyk PT, Gledhill N, Shephard RJ (2000). The economic burden of physical inactivity in Canada. CMAJ.

[15] Fleshner M (2005). Physical activity and stress resistance: sympathetic nervous system adaptations prevent stress-induced immunosuppression. Exerc Sport Sci Rev.

[16] Elmer PJ, Obarzanek E, Vollmer WM, Simons-Morton D, Stevens VJ, Young DR (2006). Effects of comprehensive lifestyle modification on diet, weight, physical fitness, and blood pressure control: 18-month results of a randomized trial. Ann Intern Med.

[17] Rozanski A, Blumenthal JA, Kaplan J (1999). Impact of psychological factors on the pathogenesis of cardiovascular disease and implications for therapy. Circulation.

[18] Björntorp P (2001). Do stress reactions cause abdominal obesity and comorbidities?. Obes Rev.

[19] Kouvonen A, Kivimaki M, Virtanen M, Pentti J, Vahtera J (2005). Work stress, smoking status, and smoking intensity: an observational study of 46,190 employees. J Epidemiol Community Health.

[20] Willi C, Bodenmann P, Ghali WA, Faris PD, Cornuz J (2007). Active smoking and the risk of type 2 diabetes: a systematic review and meta-analysis. JAMA.

[21] Flegal KM (2007). The effects of changes in smoking prevalence on obesity prevalence in the United States. Am J Public Health.

[22] Haddix AC, Teutsch SM, Corso PS (2003). Prevention effectiveness: a guide to decision analysis and economic evaluation.

[23] Thom T, Haase N, Rosamond W, Howard VJ, Rumsfeld J, Manolio T (2006). Heart disease and stroke statistics — 2006 update: a report from the American Heart Association Statistics Committee and Stroke Statistics Subcommittee. Circulation.

[24] Mendez D, Warner KE, Courant PN (1998). Has smoking cessation ceased? Expected trends in the prevalence of smoking in the United States. Am J Epidemiol.

[25] Sloan FA, Ostermann J, Conover C, Taylor DH, Picone G (2004). The price of smoking.

[26] Homer J, Milstein B, Dietz W, Buchner D, Majestic E Obesity population dynamics: exploring historical growth and plausible futures in the US. Paper presented at 24th International System Dynamics Conference.

[27] Asch SM, Kerr EA, Keesey J, Adams JL, Setodji CM, Malik S (2006). Who is at greatest risk for receiving poor-quality health care?. New Engl J Med.

[28] Russell MW, Huse DM, Drowns S, Hamel EC, Hartz SC (1998). Direct medical costs of coronary artery disease in the United States. Am J Cardiol.

[29] Sasser AC, Rousculp MD, Birnbaum HG, Oster EF, Lufkin E, Mallet D (2005). Economic burden of osteoporosis, breast cancer, and cardiovascular disease among postmenopausal women in an employed population. Womens Health Issues.

[30] Flegal KM, Graubard BI, Williamson DF, Gail MH (2007). Cause-specific excess deaths associated with underweight, overweight, and obesity. JAMA.

[31] American Diabetes Association (2003). Economic costs of diabetes in the US in 2002. Diabetes Care.

[32] Clausen J, Jensen G (1992). Blood pressure and mortality: an epidemiological survey with 10 years follow-up. J Hum Hypertens.

[33] Homer J (2008). A dynamic model of cardiovascular risk: reference guide for model version 8m.

[34] Sjostrom L, Lindroos AK, Peltonen M, Torgerson J, Bouchard C, Carlsson B (2004). Lifestyle, diabetes, and cardiovascular risk factors 10 years after bariatric surgery. N Engl J Med.

[35] Magnus P, Beaglehole R (2001). The real contribution of the major risk factors to the coronary epidemics: time to end the "only-50%" myth. Arch Intern Med.

[36] Russell LB (2009). Preventing chronic disease: an important investment, but don't count on cost savings. Health Aff (Millwood).

[37] Kahn R, Robertson RM, Smith R, Eddy D (2008). The impact of prevention on reducing the burden of cardiovascular disease. Circulation.

